# Endoscopic ultrasound-guided hepaticogastrostomy using a novel double-lumen cannula designed for a 0.018-inch guidewire

**DOI:** 10.1055/a-2791-4681

**Published:** 2026-02-13

**Authors:** Haruo Miwa, Ritsuko Oishi, Shotaro Tsunoda, Kazuki Endo, Yuichi Suzuki, Hiromi Tsuchiya, Shin Maeda

**Affiliations:** 1 26437 Gastroenterological Center, Yokohama City University Medical Center, Yokohama, Japan; 2 Department of Gastroenterology, Yokohama City University Graduate School of Medicine, Yokohama, Japan

Endoscopic ultrasound-guided hepaticogastrostomy (EUS-HGS) using a 22-gauge needle and a 0.018-inch guidewire is well suited for bile duct puncture and initial guidewire insertion 1 2 ; however, subsequent tract dilation and stent delivery remain technically challenging. Although dilation devices compatible with a 0.018-inch guidewire have been reported 3 4 , tract dilation carries a potential risk of bile leakage. Furthermore, because no catheter specifically designed for a 0.018-inch guidewire has been available, switching to a stiff guidewire generally requires multiple device exchanges. This additional step may prolong the procedure and increase the risk of bile leakage 5 .

**Fig. 1:**
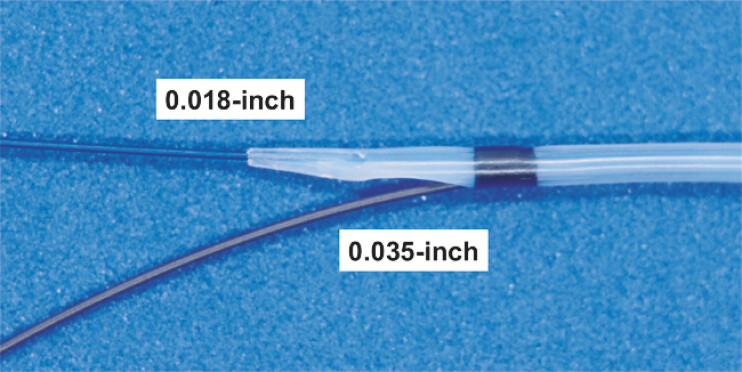
A novel uneven double-lumen cannula (UDLC); PIOLAX, Tokyo, Japan) featuring an ultra-tapered tip designed for a 0.018-inch guidewire and a side lumen compatible with a 0.035-inch guidewire.

A novel uneven double-lumen cannula (UDLC; PIOLAX, Tokyo, Japan) features an ultra-tapered tip designed for a 0.018-inch guidewire and a side lumen for a 0.035-inch guidewire, with a maximum diameter of 6-Fr ( Fig. 1 ). This design enables effective bile aspiration while allowing one-step insertion of an additional stiff guidewire without device exchange, thereby improving procedural safety and stability while minimizing tract dilation.

**Fig. 2:**
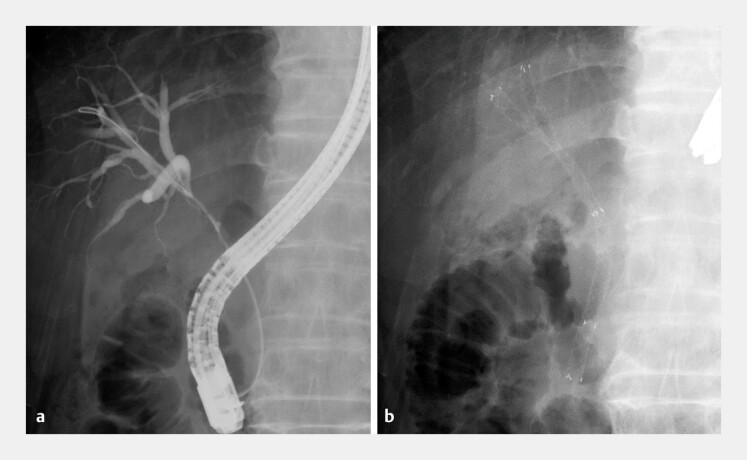
Initial drainage for hilar biliary obstruction caused by unresectable gallbladder cancer. **a** Cholangiography reveals a bismuth type IIIa stricture. **b** Fully covered multi-hole metallic stents are deployed in the right anterior and posterior branches.

**Fig. 3:**
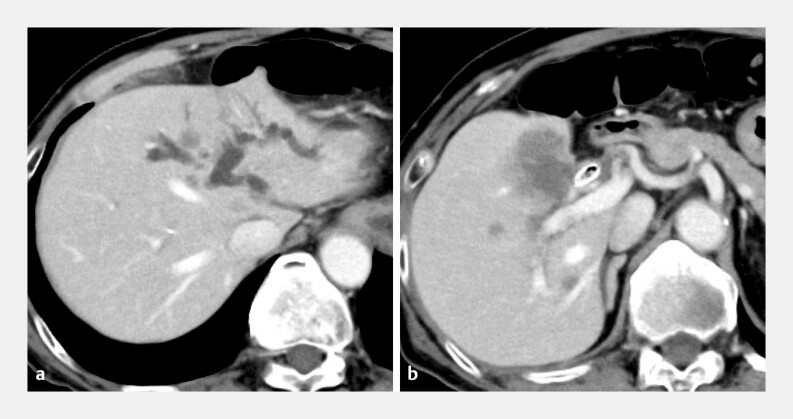
Computed tomography findings before endoscopic ultrasonography-guided hepaticogastrostomy. **a** Markedly dilation of the intrahepatic bile duct in the left lobe. **b** Gallbladder cancer with invasion of the perihilar bile duct.

**Fig. 4:**
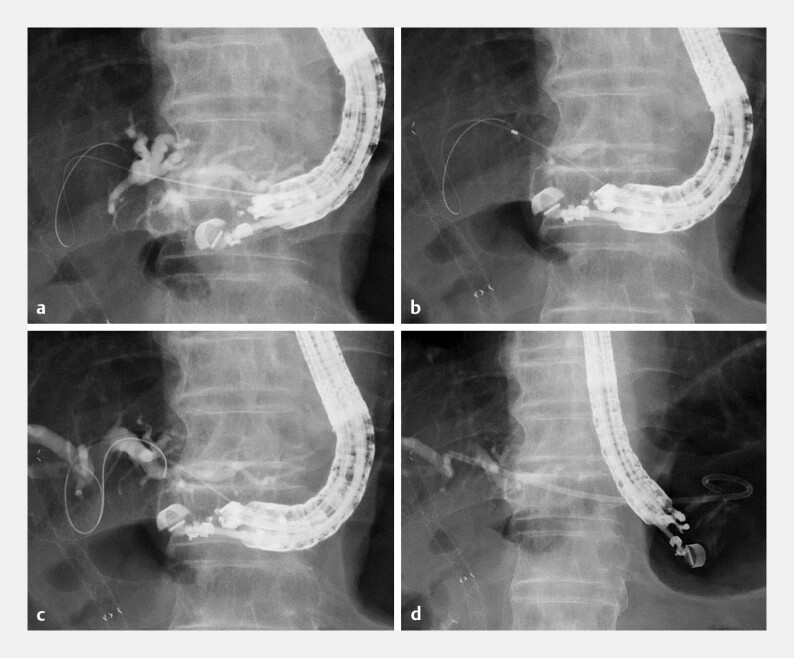
Endoscopic ultrasound-guided hepaticogastrostomy. **a** The intrahepatic bile duct (B3) is punctured with a 22-gauge needle, followed by a 0.018-inch guidewire. **b** The novel uneven double-lumen cannula is advanced smoothly, and bile aspiration is performed. **c** A 0.035-inch guidewire is inserted through the side lumen. **d** A 7-Fr dedicated plastic stent is successfully deployed.

In the present case of EUS-HGS performed in a patient with hilar biliary obstruction and previously placed fully covered multi-hole metallic stents ( Fig. 2 and Fig. 3 ), a 0.018-inch guidewire (J-wire Premier Non-marker, J-Mit Co., Ltd, Kyoto, Japan) could not be advanced across the stents. Because of the short insertion length, a 0.018-inch guidewire alone was insufficient to provide adequate stability for stent delivery. Consequently, the novel UDLC was inserted over the 0.018-inch guidewire, enabling bile aspiration followed by insertion of an additional 0.035-inch stiff guidewire. Finally, a dedicated plastic stent (7-Fr, 10 cm, Through & Pass Type IT, Gadelius Medical, Tokyo, Japan) was successfully deployed without additional tract dilation ( Fig. 4 ; Video 1 ).

EUS-guided hepaticogastrostomy using a novel uneven double-lumen cannula enabling bile aspiration and insertion of an additional stiff guidewire when advancement across pre-existing metallic stents is not feasible. EUS, endoscopic ultrasound.Video 1

To the best of our knowledge, this is the first report of EUS-HGS using the novel UDLC designed for a 0.018-inch guidewire. This device addresses the limitation associated with 22-gauge needle access during EUS-HGS.

Endoscopy_UCTN_Code_TTT_1AR_2AZ Endoscopy_UCTN_Code_CCL_1AZ_2AC
